# Factors Influencing Background Incidence Rate Calculation: Systematic Empirical Evaluation Across an International Network of Observational Databases

**DOI:** 10.3389/fphar.2022.814198

**Published:** 2022-04-26

**Authors:** Anna Ostropolets, Xintong Li, Rupa Makadia, Gowtham Rao, Peter R. Rijnbeek, Talita Duarte-Salles, Anthony G. Sena, Azza Shaoibi, Marc A. Suchard, Patrick B. Ryan, Daniel Prieto-Alhambra, George Hripcsak

**Affiliations:** ^1^ Columbia University Medical Center, New York, NY, United States; ^2^ Centre for Statistics in Medicine, NDORMS, University of Oxford, Oxford, United Kingdom; ^3^ Janssen Research and Development, Titusville, NJ, United States; ^4^ Department of Medical Informatics, Erasmus University Medical Center, Rotterdam, Netherlands; ^5^ Fundacio Institut Universitari per a la Recerca a L’Atencio Primaria de Salut Jordi Gol i Gurina (IDIAPJGol), Barcelona, Spain; ^6^ Department of Biostatistics, Fielding School of Public Health, University of California, Los Angeles, Los Angeles, CA, United States; ^7^ Department of Human Genetics, David Geffen School of Medicine at UCLA, University of California, Los Angeles, Los Angeles, CA, United States; ^8^ New York-Presbyterian Hospital, New York, NY, United States

**Keywords:** SARS-CoV-2, COVID-19, vaccine, adverse events, incidence rates, background rates, sensitivity analysis

## Abstract

**Objective:** Background incidence rates are routinely used in safety studies to evaluate an association of an exposure and outcome. Systematic research on sensitivity of rates to the choice of the study parameters is lacking.

**Materials and Methods:** We used 12 data sources to systematically examine the influence of age, race, sex, database, time-at-risk, season and year, prior observation and clean window on incidence rates using 15 adverse events of special interest for COVID-19 vaccines as an example. For binary comparisons we calculated incidence rate ratios and performed random-effect meta-analysis.

**Results:** We observed a wide variation of background rates that goes well beyond age and database effects previously observed. While rates vary up to a factor of 1,000 across age groups, even after adjusting for age and sex, the study showed residual bias due to the other parameters. Rates were highly influenced by the choice of anchoring (e.g., health visit, vaccination, or arbitrary date) for the time-at-risk start*.* Anchoring on a healthcare encounter yielded higher incidence comparing to a random date, especially for short time-at-risk. Incidence rates were highly influenced by the choice of the database (varying by up to a factor of 100), clean window choice and time-at-risk duration, and less so by secular or seasonal trends.

**Conclusion:** Comparing background to observed rates requires appropriate adjustment and careful time-at-risk start and duration choice. Results should be interpreted in the context of study parameter choices.

## Introduction

Observational healthcare data can enable large-scale medical product safety monitoring by detecting a possible rise in the incidence of adverse events following exposure. One approach commonly used in vaccine surveillance is to compare the observed incidence of adverse events following vaccination with the background incidence in the target population (Black et al., 2009). It requires accurate capture of baseline incidence rates (IRs) which becomes especially relevant for safety monitoring in new patient populations or mass preventative measures such as vaccination campaigns (Black et al., 2009; Spronk et al., 2019). While baseline IRs are commonly calculated in observational studies, there is insufficient empirical study of factors influencing incidence estimation and the magnitude of such influence, which may lead to biased inference about vaccine or drug safety.

There is no common framework to assess baseline IRs in drug safety and effectiveness studies, which results in high heterogeneity of IRs in most of the meta-analyses of IRs (Susantitaphong et al., 2013; Umasunthar et al., 2013; Hirsch et al., 2016; Dasgupta et al., 2020). Many safety studies rely on the same data source to estimate both the background and observed incidence (Spronk et al., 2019). They hypothesize that the target population used to estimate the background incidence is generalizable to the patients exposed. Nevertheless, retrospective observational studies are oftentimes performed on data sources that capture heterogeneous populations. It is unclear to what extent such populations can serve as a proxy for a counterfactual of the exposed population and whether such deviation between the comparator and that counterfactual represents a potential bias.

Patient characteristics such as age (Lin et al., 2011; Sejvar et al., 2011; Dodd et al., 2018; Willame et al., 2021), sex (Linn et al., 1996; Hanratty, 2000; Gracia Gutiérrez et al., 2020; Willame et al., 2021), race (Kanaya et al., 2011; Idrees et al., 2018; Huang et al., 2020), patient location (Linn et al., 1996; Dodd et al., 2018; Idrees et al., 2018; Marty et al., 2018) and primary healthcare institution (Beghi et al., 2011; Lin et al., 2011; Cologne et al., 2019; Willame et al., 2021) have been shown to have an impact on the IRs. For example, the studies reported up to a 10-fold difference in IRs of adverse events in different age groups (Black et al., 2009), up to a 20-fold difference in IRs across different data sources (Willame et al., 2021). Nevertheless, the influence of patient characteristics has not been studied systematically.

There is also a lack of research on the impact of time-at-risk (TAR) start and duration choice on baseline IRs. While the TAR start and duration for the intervention group is usually based on the pharmacokinetics and pharmacodynamics of the drug, they are often compared to long times-at-risk in the baseline population, and the impact of this choice is unclear. Another gap in research is related to the starting point used to estimate baseline IRs. Most of the studies use an arbitrary calendar date for time-at-risk start, which can be the date patients satisfy the inclusion criteria or start of the year for annual IRs. On the other hand, anchoring (i.e., indexing) time-at-risk intervals on a healthcare encounter may be associated with observing more adverse events due to the impact of administered drugs or detection bias.

With the ongoing COVID-19 vaccination campaign, several regulatory bodies have published protocols to assess background rates, which differ in data sources used, requirements for prior observation periods, anchoring date and outcome definitions (European Network of Centres for Pharmacoepidemiology and Phamacovigilance, 2020; Food and Drug Administration and Center for Biologics Evaluation and Research (CBER) Biologics Effectiveness and Safety (BEST) Initiative, 2020). Recent papers on estimating background rates of adverse events of interest for COVID-19 vaccine also used heterogeneous definitions and settings (Black et al., 2021; Burn et al., 2021; Nasreen et al., 2021, 19). Such discrepancies may result in producing different incidence rates and obscure their interpretation. We previously reported high variation in background rates of adverse events of special interest across age and gender (Li et al., 2021). In this paper, we systematically analyze the parameters influencing background rate estimation and discuss implications for interpreting incidence rates using the incidence rates for adverse events of special interest for COVID-19 vaccines as an example.

## Materials and Methods

**FIGURE 1:**
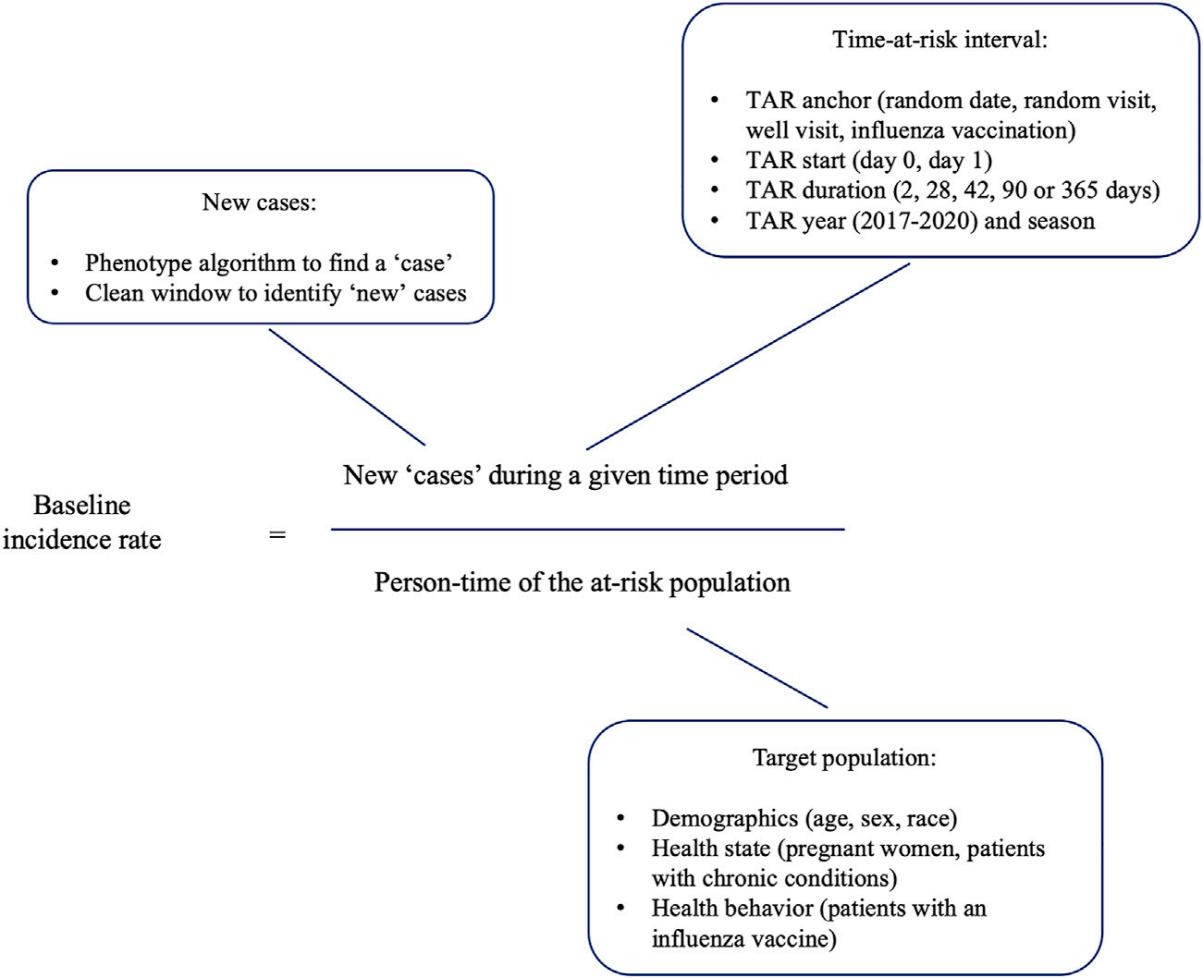
Baseline incidence rate calculation and its elements.

Our primary research question was: “How does the selection of analysis parameter choices (such as target population, anchoring event, time-at-risk, and data source) influence baseline incidence rate estimation?” To address it, we identified the set of choices related to each part of the incidence rate estimation (Figure 1) and specified experiments to estimate the sensitivity to those parameter choices.

### Data Sources

We conducted the experiment on 12 data sources (Supplementary Table S1), including sources with different data source provenance (administrative claims data, electronic health record data), origin (the US, Australia, Germany, France, Japan, the United Kingdom ), and representing different populations [privately insured employed patients in IBM MarketScan Commercial Claims (CCAE) or patients with limited income in IBM MarketScan Multi-state Medicaid (MDCD)].

### Phenotype Development

We used the outcomes (Supplementary Table S2) outlined in the “Background Rates of Adverse Events of Special Interest for COVID-19 Vaccine Safety Monitoring” protocol published by Food and Drug Administration Center for Biologics Evaluation and Research (Food and Drug Administration and Center for Biologics Evaluation and Research (CBER) Biologics Effectiveness and Safety (BEST) Initiative, 2020). The details of phenotype development were described elsewhere (Li et al., 2021).

Briefly, we followed OHDSI phenotype development and evaluation pipeline to translate and expand the phenotype definitions from the above-mentioned protocol to ensure that the clinical codes cover US and non-US data sources. This was done through translating the source codes to the standard representation in the OMOP Standardized Vocabularies (SNOMED, RxNorm and LOINC codes) and iteratively expanding the code sets using the data on code utilization in the OHDSI Network using OHDSI tool PHenotype Observed Entity Baseline Endorsements (PHOEBE) (PHOEBE, 2022). We systematically examined each cohort to assess patients’ characteristics such as demographics, baseline co-morbidities, drug use, procedures and health utilization as well as the actual codes found in the data triggering the various rules in the cohort definitions using CohortDiagnostics (CohortDiagnostics, 2022).

We did not examine phenotypes requiring an inpatient encounter on the outpatient data sources (IQVIA Australia, IQVIA Germany, IQVIA France, ICPI Netherlands). We also excluded the phenotypes that did not yield patients on given data sources, as well as age strata less than 55 years for MDCR. Results for transverse myelitis in JMDC and narcolepsy in Optum EHR were removed due to failed cohort diagnostics.

### Target Population

The base population was the patients observed in the database at any time during 2017–2019 with at least 365 days of prior observation. We also selected several subgroups of interest for COVID-19 vaccine based on health state and behavior (Figure 1). For patients with a well visit, the latter was defined as a healthcare encounter associated with CPT4 codes representing well visits. A chronic condition visit was defined as a healthcare encounter with at least one condition diagnostic code associated with a higher risk of complications as defined by CDC (Supplementary Table S2). Pregnancy episodes were constructed using a published algorithm (Matcho et al., 2018). The populations were further stratified on age (0–5, 6–17, 18–35, 36–55, 56–64, 65–74, 74–85, >85), sex (male, female) and race (White, Black). Race was extracted from the patients’ electronic health record (CUMC EHR and Optum EHR) or commercial claims (Optum SES) for whom a race field was populated.

### Time-at-Risk

We anchored the time-at-risk on a random date, health care visit, well visit or influenza vaccination, and we applied several time-at-risk interval durations (Figure 1). We studied years 2017, 2018, 2019 and 2020 separately, and we studied seasonal intervals as dates 1/1–3/31, 4/1–6/30, 7/1–9/30 and 10/1–12/31 in each year. We also compared the COVID-19 pandemic (4/1/2020–9/31/2020), to the same period in 2019.

### Sensitivity Experiment

We performed calculations for each combination of outcome, target population and time-at-risk. We calculated incidence rate as the ratio of the number of cases to the total person-time the population was at risk (from cohort start date to the end of time-at-risk period, occurrence of an outcome or loss to follow-up whichever comes first).

To make comparisons between the incidence rates observed under different analysis settings, incidence rate ratios (IRR) were computed, holding all parameters constant except for the target parameter of interest. Comparisons using IRR included: male versus female patients, White versus Black patients, no ‘at risk’ comorbid condition versus ≥ 1 “at risk” comorbid condition, outcome-specific clean window (minimum time between outcome occurrences to be considered separate events) versus no prior events as well as comparisons of different years and seasons. For all incidence rate ratios, we conducted random-effects model meta-analyses to generate age-adjusted and unadjusted pooled IRRs and 95% confidence intervals across data sources using R package metafor version 2.4 (Viechtbauer, 2010). Heterogeneity was assessed using the I^2^ index (Huedo-Medina et al., 2006). Detailed descriptions of analysis parameters for each experiment and result can be found on GitHub (Covid-19 Vaccine AESI Incidence Characterization protocol, 2021).

## Results

The number of included patients varied from 252,212 in IQVIA Australia to 40,955,085 in OPTUM EHR with the proportion of female patients from 45.0% in JMDC to 59.5% in CUMC (Supplementary Table S3). The data sources covered all age groups except for patients over 75 in CCAE and patients under 65 in MDCR with patients aged 35–54 years being the most common group.

**FIGURE 2:**
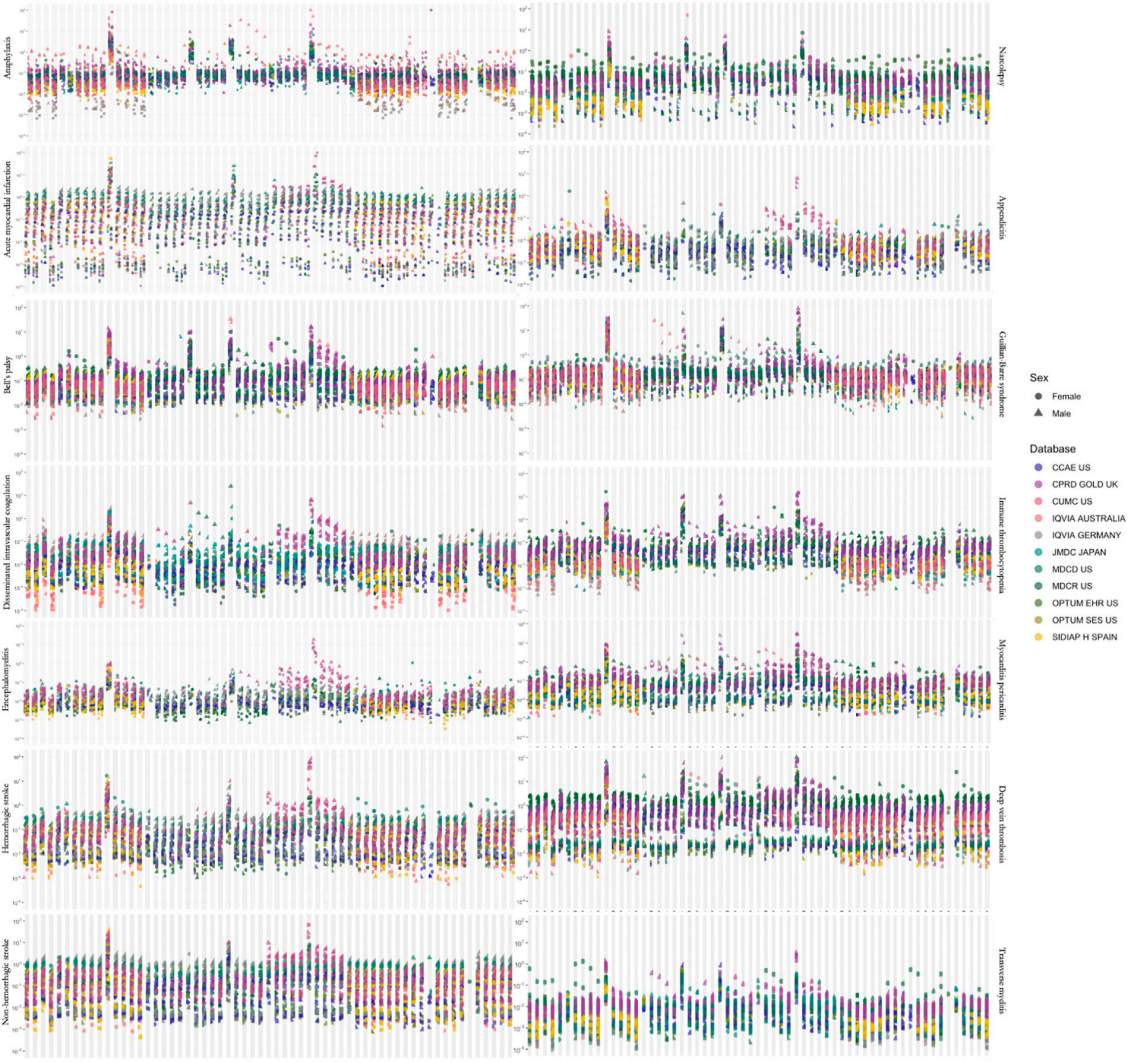
Estimated incidence rates for adverse events of interest across all **(A)** target populations, **(B)** time-at-risk intervals and **(C)** age groups. A dot represents one incidence rate estimate.

As expected, the incidence rates of the outcomes displayed a very wide range. When calculated for all age groups, target populations and anchoring events, IRs of outcomes showed more than 100,000-fold differences (Figure 2).

### Patient Characteristics

**FIGURE 3:**
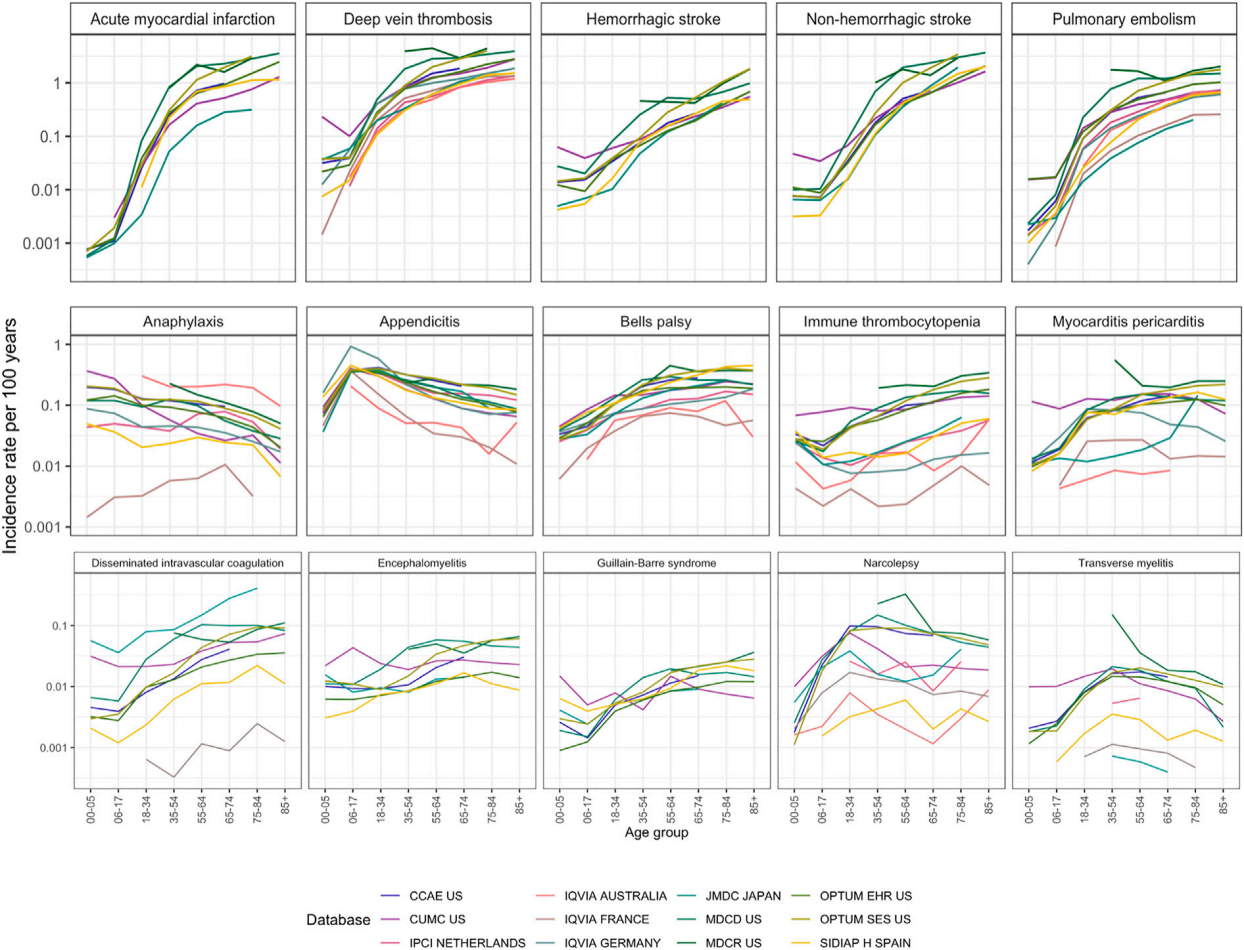
Incidence rates in age groups in 2017–2019 in patients entering on January 1 with a 365 days time-at-risk and 365 days of pre-entry observation period. Outcomes were arranged by maximum incidence per age stratum from the most common to the least common.

Age was the main contributor to the heterogeneity shown in Figure 3, with rates varying by up to a factor of 1,000 across age groups within one database. The effect of age was observed consistently across all data sources and outcomes, which highlights the extreme sensitivity of the incidence rate estimation to the age distribution of the measured population.

For sex, the IRR of incidence rates in males compared to females ranged from 0.76 to 2.17 and was statistically significant in 10 of 15 (Supplementary Table S4). The direction generally matched the literature: transverse myelitis was more common in females, cardiovascular conditions and appendicitis were more common in males.

For most of the conditions, race did not have a substantial effect on incidence rates (Supplementary Table S4 and Supplementary Figure S2, range 0.67–1.49). Disseminated intravascular coagulation, myocarditis, non-hemorrhagic stroke and pulmonary embolism were diagnosed more often in Black patients and appendicitis and Guillain-Barre syndrome were diagnosed more often in White patients.

Figure 3 also shows the database variation. Differences of a factor of 10 were common, especially for rare disorders like disseminated intravascular coagulation or transverse myelitis. Generally, these disorders had higher incidence in the non-US data sources compared to the US data sources. Notably, disseminated intravascular coagulation had a higher incidence in Japan. All age-sex population strata showed at least 40% heterogeneity by I^2^ in strata- and outcome-specific meta-analyses.

Patients with chronic conditions had significantly higher rates of all outcomes when compared to the group of patients with no chronic conditions (pooled IRR 2.16, 95% CI 1.91–2.44). Prior influenza vaccination was also associated with higher incidence compared to the general population (pooled IRR 1.41, 95% CI 1.30–1.54, Supplementary Table S10 and Supplementary Figure S8).

### Time-at-Risk

**FIGURE 4:**
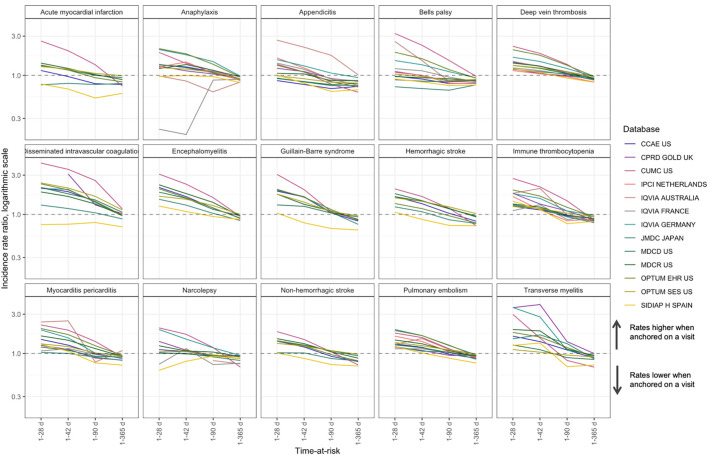
Comparison of anchoring on a random visit versus anchoring on January 1st in patients with a visit in the next year for time-at-risk 1–28, 1–42, 1–90 and 1–365 days, incidence rate ratio.

When adjusted for age, anchoring was the second-largest effect, where anchoring on a visit versus anchoring on January 1st for a short time-at-risk (2 days) was associated with up to a 100-fold increase in incidence (pooled IRR 26.8 (95% CI 21.9-32.8)). The effect was attenuated for longer times at risk (Figure 4): for example, IRR for 1–28 days was 1.4 (95% CI 1.3-1.5, Supplementary Table S5).

Additionally, we found that when anchoring on a visit, the incidence rates for a 1–365 days time-at-risk were lower than in the group of patients with a visit in the next year anchored on January 1st. This may be explained by the fact that anchoring on a visit excludes the day of the visit from time-at-risk, while time-at-risk for anchoring on January 1st includes the days of subsequent visits. Including day 0 in time-at-risk mitigates this difference (Supplementary Table S5).

We observed similar trends for anchoring on a well visit or an influenza vaccination with the pooled IRR 1.21 (95% CI 1.11-1.31) and 1.17 (95% CI 1.11-1.22) respectively (Supplementary Tables S5,S6, Supplementary Figures S4,S5). Notably, incidence of Guillain-Barre syndrome was significantly increased when anchoring on an influenza vaccination and was less influenced by anchoring on a well visit or a random visit.

Time-at-risk duration influenced incidence only when we anchored on an event. When anchoring on January 1st, comparing the time-at-risk for 1 day versus 365 days showed consistently little effect across all outcomes with the pooled IRR across databases and outcomes of 1.0 (95% CI 0.93-1.08).

We observed seasonal trends for anaphylaxis, appendicitis, acute myocardial infarction, strokes and Guillain-Barre syndrome (Supplementary Figure S6 and Supplementary Table S8). We also found a decrease in IRs in some of the data sources in 2020 compared to 2019–2017 (Supplementary Figure S7 and Supplementary Table S9).

### Incident Cases

In this study, we defined incident cases as those that occurred for the first time in a given window. An alternative approach—using all patient history to identify incident cases—produced consistently smaller incidence rates for all outcomes with the pooled IRR of 0.83 (95% CI 0.79–0.87). Notably, IRRs for narcolepsy and Guillain-Barre syndrome were significantly smaller (IRR 0.69 (95% CI 0.65-0.74) and IRR 0.59 (95% CI 0.48-0.71) respectively, Supplementary Table S11 and Supplementary Figure S9).

This observation was supported by modestly lower incidence when requiring patients to have prior observation (pooled IRR 0.94 (95% CI 0.9–0.99)). While this trend was not observed for all outcomes, narcolepsy, Guillain-Barre syndrome and myocarditis again were greatly impacted (Supplementary Table S11 and Supplementary Figure S9).

## Discussion

In this study, we observed a wide variation of incidence rates depending on the study parameters. Population characteristics had the largest impact. Even after adjusting for age and sex, the study showed variation due to the other parameters. Anchoring on any type of healthcare encounter yielded higher incidence when compared to anchoring on a random date, especially for the short time-at-risk. Duration of time-at-risk intervals showed higher rates with shorter intervals. When incident cases were defined using all patient history as opposed to pre-defined clean windows, observed incidence rates were higher.

Post-marketing safety surveillance aims at monitoring previously unrecognized serious events following medical product exposure. Active surveillance is especially relevant in the context of COVID-19 vaccination, where large populations are being exposed in a relatively short duration, heightening the need to detect a possible rise in the incidence of adverse events in a timely manner. As observed rates of events are compared to the background incidence in a population assessing causality requires accurate identification of background rates (Black et al., 2009; Spronk et al., 2019), which, in turn, depends on study parameter choices. In any observed vs. expected comparison, the comparator serves as a proxy for a counterfactual of the exposed population—what would have happened to those same individuals had they not been exposed—and any deviation between the comparator and that counterfactual represents a potential bias. In the context of safety studies, some of the above-mentioned factors can be adjusted for in the analysis, while others have to be accounted for in study design.

### Population at Risk: Age, Sex, Race

Age and sex are the key characteristics previously shown to influence IRs (Sejvar et al., 2011; Fairweather et al., 2013; Koopman et al., 2013; Hense et al., 2014; Barker-Collo et al., 2015; Dodd et al., 2018; Wang et al., 2019; Li et al., 2021; Willame et al., 2021). Our study systematically explores them and shows the extreme size of the age effect in all outcomes and data sources. Therefore, one must perform age and sex adjustment when comparing background and observed rates.

### Database Effects

The large effect of data source choice is likely a combination of actual population differences—age, sex, race, acuity, differences in genetics and environmental exposure—as well as differences in measurement, such as collection via administrative claims versus electronic health records. Some data sources may be appropriate only for certain conditions due to their population characteristics. For example, MDCR contains patients over 65 years old, which makes it a poor choice for studying pediatric conditions. Data sources that reflect only some aspects of care (such as outpatient data sources like IQVIA Australia or IQVIA Germany) may yield different rates for conditions that commonly require hospitalization. The differences suggest that, where possible, background rates should be calculated in the database where the surveillance will be done. Where this is not possible, a broad range of databases should be used and, based on a random-effects meta-analysis, prediction intervals should be calculated for the incidence rates. We demonstrate our prediction intervals in Supplementary Table S12.

### Large Effect of Anchoring on Health Encounters

Anchoring was the second most important parameter to be accounted for, at least at the shortest time-at-risk. Its influence was not quantified before and, surprisingly, was present for both random and well visits.

When studying background incidence in the context of COVID-19 vaccination (in cohort or self-controlled studies), estimation of IRs of events following vaccination is anchored on the date of vaccination. To appropriately compare it to the background rates, one has to make an assumption of the type of encounter that represents the vaccination best. For example, in a wide population that receives the vaccine based on availability, a random date may be a good approximation for the date of vaccination. On the other hand, vaccination date in patients receiving vaccine upon hospital discharge or in nursing homes may represent a strong anchor with the effect like or even greater than anchoring on a random visit. This is especially relevant for outcomes like anaphylaxis with short times-at-risk.

Influenza vaccination may serve as another proxy for COVID-19 vaccination, in terms of defining an anchoring event. But the population that receives an influenza vaccine in healthcare institutions may be different from those who receive it in pharmacies (Drozd et al., 2017). It may explain why we observed higher incidence of conditions in patients with prior influenza vaccine as vaccination in this case may be indicative of co-morbid conditions.

### Muted Seasonal Effect and Small Annual Increase

While previous research emphasized the influence of season on IRs (Marrero et al., 2016), we observed that seasons had a minor effect on incidence. The direction of difference we observed generally matched the literature (Nagarajan et al., 2017; Dodd et al., 2018; Chaaban et al., 2019; Tschöpe et al., 2021). Temporal trends were moderate: incidence rates slightly increase from 2017 to 2019, which may correspond to better diagnosis or changes in coding practices. That agrees with the findings in the literature for encephalomyelitis, hemorrhagic stroke, anaphylaxis, narcolepsy, Bell’s palsy (Kadambari et al., 2014; Katan and Luft, 2018; Chaaban et al., 2019; Hamedani et al., 2021; Lee and Kim, 2021; Willame et al., 2021).

### Incident Cases

The strategy for selecting a clean window (minimum time between outcomes) consistently influences background rates. Lower incidence of chronic conditions or conditions that are likely to occur once (such as appendicitis) when using narrow clean windows reflects potential index event misclassification. It is possible that such patients are captured later in the course of the disease, which requires thoughtful examination of the patient history to determine the true condition start date.

Using a requirement of prior observation ensures that patients were actively observed in the data source. In this study, we found that such a requirement did not produce a difference in IRs when compared to the broad population. On the other hand, it potentially reduces index event misclassification as more information about the patient is captured.

## Limitations

Due to observational nature of the study, the data sources may not have complete capture of patient conditions. While our phenotype algorithms may be subject to measurement error, such error in unlikely to be differential. As the goal of the study was not to establish causality but to estimate sensitivity of incidence rates, phenotype measurement error or partial data capture should not influence the results of the study. As race is available only in three US data sources, our findings regarding race influence may not be generalizable to other data sources or populations. Additionally, in the data sources we extracted the race from, the latter is ambiguous due the different setting, the person collecting it, and the reason for the collection. Differences in incidence of adverse events of interest in different races may be attributable to differences in healthcare utilization, clinical presentation and health state awareness rather than a true difference in incidence.

## Conclusion

Accurate estimation of background rates is essential for their use in safety or effectiveness studies. Background incidence rates are highly sensitive to demographic characteristics of population, so estimation requires age, sex, and potentially other adjustments, and they would best be performed within the same database. Even when adjusted for these factors, incidence rates are highly influenced by the choice of the time-at-risk start date or event. When comparing background rates to estimated incidence rates, one must examine if the choice of anchoring is compatible between groups. If anchored, short time-at-risk intervals are associated with higher incidence, so the choice of time-at-risk requires thoughtful analysis. Similarly, the choice of clean window for defining incidence cases results in different incident rates. Finally, the choice of year and season may influence rates, albeit the influence is not prominent compared to the other factors.

## Data Availability

The original contributions presented in the study are publicly available. This data can be found here: https://github.com/ohdsi-studies/Covid19VaccineAesiIncidenceCharacterization/blob/master/extras/incidence_rates_sensitivity_experiment_results_060121.csv. The protocol is available from https://github.com/ohdsi-studies/Covid19VaccineAesiIncidenceCharacterization/raw/master/extras/Vaccine_safety_sensitivity_protocol.pdf.
